# Virtual home visits during COVID-19 pandemic: mothers’ and home visitors’ perspectives

**DOI:** 10.1186/s12884-023-05896-9

**Published:** 2023-08-11

**Authors:** Abdullah Al-Taiar, Michele A. Kekeh, Stephanie Ewers, Amy L. Prusinski, Kimberly J. Alombro, Nancy Welch

**Affiliations:** 1 School of Community & Environmental Health, College of Health Sciences, Old Dominion University, 3136 Health Sciences Building, 4608 Hampton Blvd, Hampton BlvdNorfolk, VA 460823508 USA; 2 Virginia Department of Health, Chesapeake Health District, 748 Battlefield Blvd North, Chesapeake, VA 23320-4941 USA

**Keywords:** Maternal, infant and early childhood home visiting, Virtual home visiting, COVID-19, Focus group discussion

## Abstract

**Background:**

The experiences of mothers enrolled in Maternal, Infant and Early Childhood Home Visiting (MIECHV) program with virtual home visiting (VHV) during the pandemic remain mostly unknown. This study aimed to describe in detail the experience of home visitors and mothers with VHV during COVID-19 pandemic. This is a prerequisite for guiding future efforts to optimize MIECHV services that are provided through virtual operation.

**Methods:**

Focus groups discussion were conducted with home visitors (*n* = 13) and mothers (*n* = 30) who were enrolled in BabyCare program in Virginia from January 2019 to June 2022. This included mothers who received in-person home visiting (IPHV), VHV, or both (hybrid IPHV and VHV). Inductive analysis was used to identify emergent themes from the transcripts, then coding was conducted following a codebook that was developed by the research team.

**Results:**

Both mothers and home visitors considered IPHV necessary for a proper assessment of developmental milestones of children, for the assessment of the growth of the child through measuring the weight and height/length of the child, for the mothers to open up and discuss sensitive issues like domestic violence, for building a relationship between home-visitor and the parents, and for other potential benefits (comprehensive assessment of the environment around the child inside and outside the house from home visitors’ perspective and detecting abnormal health conditions in children from mothers’ perspective). Both mothers and home visitors see that VHV has some role to play but not to be a replacement for IPHV. If VHV is to be used, video conference is preferred by both mothers and home visitors, as it allows for some assessment.

**Conclusion:**

Mothers and nurses considered IPHV critical for proper and comprehensive assessment of the child and the family and also essential to build the nurse-client relationship. Both mothers and home visitors considered VHV supplementary to IPHV that can be used from time to time particularly with busy mothers. VHV may have little room with parents with intellectual disabilities and the difficulty in dealing with technology seems to be no longer a major issue.

**Supplementary Information:**

The online version contains supplementary material available at 10.1186/s12884-023-05896-9.

## Background

The Maternal, Infant and Early Childhood Home Visiting (MIECHV) program was developed to support home visits for pregnant women and families with children up to kindergarten entry targeting those who are living in communities at risk for poor maternal and child health outcomes [1]. The MIECHV program is administered by the Health Resources and Services Administration (HRSA) in partnership with the Administration for Children and Families. States, territories, and tribal entities are funded to develop and implement home visiting programs based on the evidence that home visits by a nurse, social worker, early childhood educator, or other trained professionals during pregnancy and early childhood improve the lives of children and families [2]. Currently, the U.S. Department of Health and Human Services recognizes 19 home visiting models as evidence-based, hence are eligible for state and territory MIECHV Program funding [3]. However, states and territories are allowed to spend up to 25% of funding to implement promising approaches/models that can be rigorously evaluated for their effectiveness. In Virginia, there are three evidence-based home visiting models including Parents as Teachers (PAT), Healthy Families America (HFA), and Nurse Family Partnership (NFP). In addition to these models, there are several promising models, one of which is the BabyCare Program.

During the COVID-19 pandemic, many home visiting programs shifted to virtual service delivery (telephone and/or video technology) to protect the health and safety of families and the home visiting workforce [4]. Currently, some states allow the use of telephone and/or video communication instead of face-to-face home visits while others are returning to in-person visits while following the CDC recommendations [4]. The impact of the shift to telehealth on the effectiveness of MIECHV programs remains mostly unknown [5]. However, a recent study in Florida found that the implementation of audio-only virtual prenatal visits was not associated with changes in perinatal outcomes and rather increased prenatal visit attendance in a vulnerable population during COVID-19 pandemic [6].

Although few studies have attempted to measure patients’ or clients’ satisfaction with virtual prenatal visits as a replacement for visiting healthcare centers during the pandemic [7, 8], no study has explored women’s experiences and perceptions of virtual home visiting (VHV) in MIECHV programs. In 2020, a study explored the experience and perceptions of MIECHV staff (not families) on VHV in Florida [9]. The investigators used a secured Qualtrics survey link to collect quantitative data along with focus group discussions to collect qualitative data. In this study, home visitors in MIECHV perceived VHV to be feasible and essential to provide support for families who may not otherwise have the means or knowledge [9]. In a recent study, authors compared the characteristics of families enrolled in one of the MIECHV programs before and after the onset of the COVID-19 pandemic using routinely collected data. They found that families enrolled after the onset of the pandemic were less likely to be impacted by housing problems, have a child with a disability, or be involved in the military but these families were more likely to have a history of child abuse or neglect [5].

A major aim of home visiting programs is to build relationships that extend beyond parenting and child development [10]. Therefore, it is critical to investigate mothers’ experiences and perceptions of VHV within MIECHV programs to elucidate the success/failure of VHV in building those relationships. In all healthcare services, it has been demonstrated that clients’ experience and satisfaction increase the compliance with treatment and recommendations [11–13], enhance treatment outcomes [14, 15], predict patient health-related behavior [16–18] as well as patient utilization of care and continuity with the same provider [19–21]. In fact, because clients’ experience and satisfaction reflect the success in meeting clients’ values and expectations, they are the most commonly used outcome indicators for quality of care [22–24]. In MIECHV programs, it is not clear if women perceive VHV as superior or inferior to in-person home visiting (IPHV) and if so, why. Moreover, there is evidence of a decline in the enrollment in some MIECHV programs after the onset of the pandemic, which raises the question of whether MIECHV programs can effectively engage families and attract mothers through VHV [5]. Therefore, exploring mothers’ perceptions and experience with VHV is essential to guide MIECHV programs in the next few years during which MIECHV programs across the nation consider the possibility of VHV either as an augmentation or a replacement for most IPHV. This study will inform MIECHV with the ultimate goal of optimizing the programs and hence achieving an optimal patient-centered care.

We used BabyCare program in Virginia, which is a promising model that has higher flexibility to explore and implement changes in VHV compared to evidence-based programs. The program began in 1992 and includes home visiting provided by a registered nurse for pregnant women and infants up to two years of age. The program provides case management by a registered nurse, nursing assessment of mother and child, screening and referrals for several conditions including substance use, depression, and intimate partner violence, as well as screening and referrals for developmental delays for infants/children. The home visiting starts with an admission visit, which includes in addition to routine paperwork, screening for behavioral health risks and screening for maternal depression as well as assessing the family needs. Then the program develops a service plan with goals to address medical, psychosocial, educational, and other concerns as well as follow up the compliance of referrals and medical appointments. The service plan may also include providing a child car seat to those who qualify, referral to smoking cessation services, referrals to WIC (to receive supplemental food, special formula, breast pumps, and breastfeeding support), providing birth control and other sexual health services for clients seen in family planning clinics, weight checks for infants and administering Tdap and influenza immunizations for pregnant women [25]. Eligibility criteria for women to be included in the program comprise women who are eligible for Medicaid and pregnant or have an infant/child aged less than 2 years, have experienced teen pregnancy or unplanned pregnancy, experiencing (or have a history of) abuse in their home, suffer from or have a history of postpartum depression. For children, eligibility criteria include diagnosis with neonatal abstinence syndrome or fetal alcohol syndrome, experience of substance exposure, referral to child protective services, failure to thrive, prematurity, low birthweight, maternal or paternal absence, or exhibition of poor emotional bonding. Families enter BabyCare services through referral from different sources including medical/health providers, early childhood programs (e.g., WIC), or other referral sources. Like all other MIECHV programs, BabyCare in Virginia suspended IPHV in March 2020 and provided telephonic and contactless drop-off services for families in the program without admitting new cases. VHV was initiated in June 2020 after obtaining the permission, training staff, and securing the necessary equipment. This was conducted through Videoconference (Zoom or Doxy.me) but sometimes through telephone calls particularly when the technology fails. The aim of this study is to evaluate home visitors’ as well as women’s experience and perceptions of VHV in BabyCare home visiting program in Virginia.

## Methods

### Study participants

The study population included home visitors and mothers who were referred to BabyCare program in Chesapeake district in the period January 2019-June 2022. This included mothers who received IPHV, VHV, or both (hybrid IPHV and VHV). Staff members from the BabyCare program informed all mothers that a research team would like to discuss their experience with home visiting before and after the onset of COVID-19 pandemic asking for mothers’ permission to provide their contact details to the research team that will provide more information about the study. After receiving the contact details of mothers, researchers called them and invited them to participate in the study. The invitation included the statement “*a* *team of researchers aims to improve women’s experience with virtual home visiting in BabyCare program and to understand their perspectives of home visiting through audio-only or videoconferencing”.* Monetary compensation was provided to the mothers and all study participants signed a written informed consent before each group discussion. The study was reviewed by Old Dominion University Institutional Review Board and was determined to be “exempt”.

### Study design and data collection

We used focus groups discussion to provide women with the opportunity to describe their experiences and perspectives regarding virtual home visits without any pre-set answers. The open-ended nature of the questions used in focus groups allows the participants to draw on different aspects of their experiences and elucidate the perceptions of participants as to the benefits and barriers of this modality of home visiting compared to traditional IPHV. Six focus groups discussion were conducted (2 with home visitors and 4 with mothers) to understand the experience, perception and perspectives of both home visitors and mothers. We used purposeful maximum variation sampling to select mothers that varied markedly from one another [26]. The aim was to explore the most diverse views and experience of VHV in the BabyCare program in the Chesapeake district. We selected mothers to participate in group discussions from those who received IPHV before COVID-19 pandemic, VHV only, and both. Within these categories, women were selected from different ethnic groups, and the type of services received (prenatal home visits or child-related home visits).

The focus groups were facilitated by a well-trained facilitator who used separate question guides for home visitors and mothers enrolled in the program. A member of the research team was responsible for the notes taking. The question guide for home visitors included general questions on the work experience and environment in the program and then questions on the transitioning from IPHV to VHV as well as experience with VHV (Table 1). The question guide for mothers included questions on their experience with the program in general and then questions on their experience with VHV as well as IPHV. Mothers were also asked about their preferences for VHV or IPHV as well as video VHV or audio only VHV (Table 2). While the focus groups discussion with home visitors were conducted at the Department of Health, focus groups with mothers were conducted at a local public library or a church. This meant to encourage mothers to speak freely and express their opinion and experience away from the service providers. Mothers were also assured that staff from BabyCare will not have access to the data and will not hear what is discussed. It was also thought that Hispanic mothers may not share their experience openly if the focus groups included mothers from other ethnic groups or because of linguistic barriers. Therefore, a separate focus group was arranged for Hispanic mothers, which was led by a native speaking facilitator and was conducted in a public library.

**Table 1:** Question guide used in focus groups discussion with home visitors

Q1) How would you describe your experience and feelings about working in BabyCare program in general?
*Probe: what factors or resources make you feel satisfied with your job at BabyCare program?*
*Probe: what factors or issues that make you unsatisfied with your job at BabyCare program?*
Q2) How would you describe your feelings/thoughts and experience during the transition from in-person home visiting to virtual home visiting which occurred due to COVID-19 pandemic?
*Probe: What was your initial feeling and experiences about the transition? *
*Probe: Have these feelings and experiences changed over time?*
*Probe: Do you feel you have adjusted with virtual home visiting?*
*Probe: What personal or job-related factors that hindered or facilitated the transition? *
Q3) How would you describe your experience with virtual home visiting in BabyCare program?
*Probe: Aside from what you said, can you think of any other advantages of virtual home visiting?*
*Probe: Aside from you said, can you think of any other problems/disadvantages of virtual home visiting?*
Q4) What did you like best about in-person home visiting?
Q5) What did you like best about virtual home visiting using the phone or zoom/doxy.me?
Q6) In your experience, what was the difference between the Audio and Video home visiting?
*Probe: Aside from what you said, can you think of any other difference between audio and video home visiting?*
Q7) Based on your experience during the last two years, if you could choose to provide in-person visit or virtual home visit, which one you would choose and why?
*Probe: Aside from you said, can you think of any other reason for this choice?*
Q8) Of all the things we’ve discussed today, what would you say are the most important issues you would like to express about virtual home visiting in BabyCare?
Q9) These are all of my questions, is there anything you would like to add that I didn’t ask?

**Table 2:** Question guide used in focus groups discussion with mothers

Q1) How would you describe your experience with BabyCare program in general?
*Probe: what makes you feel satisfied with services provided by BabyCare program?*
*Probe: is there anything makes you unsatisfied with services provided by BabyCare program?*
Q2) How would you describe your experience with virtual home visiting (by phone or video) in BabyCare program?
*Probe: Aside from what you said, can you think of any other advantages of virtual home visiting?*
*Probe: Aside from what you said, can you think of any other problems/disadvantages of virtual home visiting?*
Q3) What did you like best about in-person home visiting?
Q4) What did you like best about virtual home visiting using the phone or zoom/doxy.me?
Q5) In your experience, what was the difference between the Audio and Video home visiting?
*Probe: Aside from what you said, can you think of any other difference between audio and video home visiting?*
Q6) Based on your experience, if you could choose to receive in-person visit or virtual home visit, which one you would choose and why?
*Probe: Aside from what you said, can you think of any other reason for this choice?*
Q7) Of all the things we’ve discussed today, what would you say are the most important issues you would like to express about virtual home visiting in BabyCare?
Q8) These are all of my questions, is there anything you would like to add that I didn’t ask?

### Data analysis

With participants’ permission, all focus groups discussion were digitally recorded, transcribed verbatim, and transcriptions were checked for accuracy. First, preliminary inductive analysis was used to identify emergent themes from the transcripts, then coding was conducted following a codebook that was developed by the research team based on the focus group guide and the emergent themes. This was done separately for group discussions with home visitors and mothers (List 1a and List 1b, respectively). All transcripts were coded by the leading investigator and another team member blind-coded all transcripts. After the initial coding, the codebook was refined, and discrepancies were reconciled. After coding all transcripts, the results were summarized based on content analysis using the coded transcripts. Finally, the written report was sent to the participants staff of the BabyCare program to solicit their feedback regarding the accuracy of the findings.

## Results

The characteristics of the mothers and staff who participated in groups discussion are shown in Table 3. The mean (SD) age of mothers was 33.3 (7.1) years, and the majority of the participants were black American. Most participants were single mothers with a level of education equal to or less than high school and household income less than $ 60,000.

**Table 3:** Characteristics of the staff and the mothers who participated in focus groups discussion

**Characteristics of staff**	
**Age (years); mean (SD)**	49.0(10.7)
**Duration of work with BabyCare program (years); median (IQR)**	4.0(4.2)
**Race**	**n (%)**
**Black American**	5(38.5)
**Hispanic**	1(7.7)
**White American**	5(38.5)
**Others**	2 (15.4)
**Characteristics of mothers**	
**Age (years); mean (SD)**	33.31(7.1)
**Race**	**n (%)**
**Black American**	14 (46.7)
**Hispanic**	11 (36.7)
**White American**	3(10.0)
**Others**	2 (6.7)
**Marital Status**	**n (%)**
**Single**	22 (73.3)
**Married**	8 (26.7)
**Household Income**	**n (%)**
**< 30,000 $**	16 (53.3)
**30,000 to < 60, 000$**	11 (36.7)
**60,000$ to < 90, 000$**	0 (0.0)
**90, 000$ to < 120,000 $**	1 (3.3)
**Prefer not to tell**	1 (3.3)
**Highest level of education**	**n (%)**
**No formal education**	2 (6.7)
**Elementary school**	3 (10.0)
**Middle school**	2 (6.7)
**High school**	15 (50.0)
**University degree**	7 (23.3)
**Prefer not to tell**	1 (3.3)
**Working in paid job**	**n (%)**
**Yes**	18 (60.0)
**No**	12 (40.0)

### Overall experience with the program

To gauge the experience of home visiting staff with VHV, it is essential to understand the overall work environment in the program from their perspective. Therefore, group discussion with home visiting staff started by asking questions about the work environment including the resources and motivations for staff. All home visiting staff expressed their affinity to their job describing the job as the right position, or the program as an exceptional or very great place to work citing multiple reasons such as the interaction with mothers, going to the community, playing with children, assessing their developmental milestones, or observing some success such as a child going back to school. Participants also enjoyed working with the program because it is challenging and requires shifting from office work to going to the field to coordinating with other agencies. Several participants cited their previous experience as the underlying reason for their decision to work in the program. For example, one participant worked previously as a school nurse and noted children at the age of five years not able to recognize common shapes or colors, which inspired her to be part of a program that teaches parents to read to their children and provide the tools for families. As one of the nurses said, *“I was like, what is missing that some of these children arrive at five years old and not having had exposure to those common shapes like circle, rectangle, red, blue, yellow, that I think a lot of us really take for granted."*

The home visiting staff were particularly satisfied with the support from their colleagues in different forms including guiding them to books or sharing their experiences in supporting families. One issue that affected their satisfaction was their inability to meet some mothers’ needs, particularly in relation to transportation and housing. While talking about their motivations regarding their work, several participants pointed out the impact of the pandemic on the aspects of the program they enjoyed in their work such as not being able to play with children and losing the connections with mothers. Another motivational factor that nurses expressed was the feeling that people trust them and welcome them in their homes, which was also affected by the pandemic. As one of the nurses described, *“And again, with the pandemic, not being able to go into the homes and hold the babies and play with them and kind of make that connection with our moms has been challenging”.*

Similarly, before the discussion of mothers’ experience with VHV, they were asked about their overall experience with the program. Most mothers described the program as “really helpful”, “awesome” or a program that met all their needs emphasizing the quality of the home visitors describing them as “very sweet”, “wonderful”, “great”, “amazing”, “very caring”, “advocate for me”, or “nonjudgmental”. Participants provided individual stories of how the program connected them to various sources to obtain their needs including clothes and food.

*“The BabyCare program has been awesome for me, helped me tremendously for real, even car seats. They put you in a car seat program if you don't have, you know what I mean, a car seat or can't afford a car seat, and things like that, or whatever, have the health department offers that. But, you know, the BabyCare will make sure everything is scheduled for you so you don't have to stress yourself into, like you said, if everything's okay.”* Mother in BabyCare program.

*“I really enjoyed the baby care program. I've had two people now I’ve been working with, and they were both sweet and amazing. My son had a tongue-tie. My son had a tongue-tie, so there was a lot of difficulty breastfeeding.”* Mother in BabyCare program.

Mothers pointed out other services that the program provided including monitoring and assessing milestones and detecting abnormal features in their children. In addition to these, some participants pointed out the value of the program as a major source of social and emotional support beyond helping mothers with their material needs in normal circumstances or when they have strained situations like postpartum depression or unplanned pregnancy. As one mother said, *“So it's nice to know that there's a person you can get support like outside of your family because sometimes you don't want to ask for help of family members and sometimes it's okay to know that you can go to someone else”*. Mothers repeatedly emphasized this point describing the nurses as one of their family members. As one mother said, *“It kind of makes me feel like I have a support system because my support system is very small. My family isn't big by any means. And then on my side of family, it's the family that I don't really want to keep in my life or my kids' life, but, you know, much less. So, it kind of feels like you have a second parent almost, like a grandparent, or like an aunt, or uncle, or whoever your BabyCare worker may be because I'm sure there's probably males in the program.”* while another mother said, *“Yeah, that's what I was going to say. With me being in there for so long, you know, my kids with the lady I have they know her very well. It is like she is family to us.”*

Mothers in different groups described with different examples how the program provided them with the opportunity to discuss their child’s illness, which guided them to the appropriate medical service when they felt they had not received a proper medical care from their clinicians. First-time mothers and mothers of children with developmental issues felt that the program is a major source of information that they would not be able to obtain from other sources.

*“.. my daughter was actually in the ER because of a stomach virus that was going around. And she was just not acting right. Like, she was sleepy all the time and everything. They sent her home because, like, "Oh, it's a stomach virus." I brought her back, and her sugar was actually 46, and they're like, "I am so sorry we sent her home. And it's because of my BabyCare worker I actually went back in because I was like, "Okay, maybe I'm just being a mom and just being overprotective." But hearing it from somebody else, from an outside opinion kind of helped me to bring her back in, and I'm really glad I did.”* Mother in BabyCare program.

*“Like it really has been awesome because without the knowledge from them, if you never even had a child... You can't just pick up the phone and call your doctor's office, and be like, "What, what can I do? What I'm supposed to do?"* Mother in BabyCare program.

*“So, I had my mother with my first two. Now I don't have her. So, I was sitting there, "How I'm supposed to take care of this baby with this kinda issue?" And she gave me all the knowledge. “* Mother of child with disability in BabyCare program.

### Impact of the pandemic on the home visiting including transitioning to virtual operations

Nurses described the transition from IPHV to VHV as “*better than doing nothing*” at that time highlighting the need of transitioning back to IPHV. Several reasons were cited for this perception including their training as nurses, which emphasizes the direct observation and the physical contact with mothers and their families during the assessment. As one of the nurses explained, *“My lens is completely physical contact with the clients and their family, their house, their everything. And so, I think it is really important that we need to transition back to home visitation, maybe with a few instances where we can do a virtual or something like that because you are busy or mom calls and says, yeah, I can't say you come today because I have to do this and that, but we can talk now we can switch it over to virtual or just talk.”*

Nurses described transitioning to VHV as challenging particularly during the period in which there was no decision about home visiting at the beginning of the pandemic. During that time, staff in the program were asked to help in other tasks such as contact tracing, and case investigation while still doing contactless drop off for materials that mothers urgently needed. When VHV was approved, it was challenging to get access to the software that helps with VHV and to train the nurses to use the technology, as well as to make mothers comfortable with it. There was a need to upgrade the old phones and provide equipment that are not covered by the Department of Health. Another major challenge was the absence of electronic medical records and that most of the assessment tools continued to be done on papers during the pandemic. Several factors facilitated the transition, particularly taking verbal consent instead of dropping paperwork for clients to sign. Some nurses particularly those who joined the program just before the pandemic described their stress as they were not confident in dealing with technology. This was alleviated by support from other team members.

*“And I'm not always the most confident when it comes to technology. So, I was trying to gain confidence in learning that. And, so for me, it was a little stressful, some excitement there but then it would go back to stress, but, like Name, a lot of the nurses have already mentioned, there's a really great support system here and we always go to one another and I feel like all of us are open to teaching each other and always have the patience or find time to guide us with whatever challenges we may encounter.”* Nurse in BabyCare*.*

Another challenge in the transition from IPHV to VHV during the pandemic was that all organizations that help mothers including shelter, food and social services changed their procedures and agendas because of the pandemic and there was a great need to update the sources of help to mothers. Overall, these have created stressful situations, which all nurses agreed resulted in a lot of learning. As one of the nurses explained, *“So every time a client has a need, there is a guide or a list that you can actually go to and try to help. Now with the pandemic, like everybody says, everything has changed. So, we have to keep it updated and try to connect with organizations to see how things have changed or the services have changed with them.”*

Group discussion with the mothers showed that mothers’ perceptions of the risk during the pandemic increased their acceptance of VHV as a safe option during the pandemic. As one mother said, *“But, I mean, for the protection of things, it was fine, I mean, you know, I'd rather us be distant, you know, so we don't get sick.”.* Several mothers confirmed they received the same attention during the pandemic from the program. Two mothers of children with disabilities described how useful home visiting was during pandemic when visiting doctors’ offices was limited. As one mother said, *“And the home visit was really good because during COVID, you know, at the time they had stopped people from going to the doctor's office. And then, by her coming by the house, watching with the growth of my daughter was really good for me. So, it was a really good experience for her coming there, showing me the growth, things I need to know. How big she should be getting. I enjoyed that.”*

### Importance of in-person home visiting (IPHV)

Throughout the discussion, nurses described in detail the importance of IPHV to fulfill the mission of the program and provide solid assistance to the mothers. They cited specific examples from their work experience highlighting how VHV would not allow them to provide that aspect of the service. Nurses and mothers highlighted the following reasons for conducting IPHV:

- IPHV is necessary for the assessment of developmental milestones, which was raised repeatedly by several nurses. Nevertheless, nurses pointed out that further training on assessing milestones virtually may help in this issue (see below). In fact, a supervisor nurse reported that several nurses managed to assess the developmental milestones through VHV using video conferencing.

*“I'll just speak from the supervisor standpoint that when I audit the charts and I see that the nurses have done and completed telehealth visits, they are documenting, you know, child is sitting, you know, saw the child sitting on the lap, well supported, clean the environment looks, you know, the home looks, clean and organized or it was a little disheveled, but mom's been sick, you know, and witnessed mom, you know, cooing at and connecting with the baby. So, there was still a lot of assessment being done and there was a lot of assessment being done and captured in their progress notes in the charting.” A supervisor nurse in BabyCare.*

Several mothers specifically referred to the issue of evaluating milestones during groups discussions while talking about the importance of IPHV as the main route to do this. Some mothers realized that the assessment might be possible through video VHV, but felt it is not complete or effective compared to the assessment during IPHV. As one mother put it *“I know they can't do like the exact milestones, but they can do some of that stuff.”.* It seems evaluating milestone is one of the major reasons for mother’s preference for IPHV.

*“Well, I prefer the in-home visits over the virtual because it's more hands-on. The in-home visits, it helped me to know that, you know, each one is meeting milestone and, they're developing right on time. It just reassures me. You know, with virtual, you can't really. You can see, but you can't really, you know, get that... I don't know how to describe it. I mean, you could see the baby crawling, but like if she crawled wrong or moving more skew to the right, you'll be able to diagnose it better “*Mother in BabyCare program.

*“So those milestones and that stuff is really super important. And if you're not getting it because you're in virtual, how could you know, you want to be the best mom do the best thing for your kids. And those are really great tools to help us with that”* Mother in BabyCare program.

*“And sometimes the same questions that they ask you on the phone, well they also make them right there. With the only advantage, as she said, is that they evaluate or weigh him, measure him and see him in the activities because on the phone they ask one all of what they can ask everything that can be asked about growth, development and all that. But, in person, well they can see because they are observing him.”* Spanish speaking Mother in BabyCare program.

b) IPHV is necessary for assessing the growth of the child through measuring the weight of the child and reporting that to the parents. This was an important aspect of the service that mothers appreciated. Nurses described that they had to rely on the parents reporting the weight of their child if they have been recently to a clinic or sometimes instructed the parents to weigh their child if they have a weight scale. As one of the nurses explained,

*“So, when we are in the home, we weigh the baby on each visit. And that was one of the things that our clients could really love to see how much their baby has grown. Like, "Oh." You know, well, they don't get that anymore. And then when you do ask them, like I don't, or if they haven't been to the doctor recently, I mean, they don't know their current weight. They know they're feeding the baby. But we don't have that way of weighing them. And that was like a huge thing for our clients. And, and I remember as a parent, you know, as a mother, I enjoyed knowing how much my baby weighed also. So, you know, I try to do, you know, creative things where if they have a scale in their home, I just tell them to weigh themselves. Okay. And then weigh themselves with a baby (laughs) and then, you know, add the difference.”* Nurse in BabyCare*.*

Groups discussion with mothers also confirmed that measuring and monitoring the weight of child is an important reason for having IPHV. This was brought up in every group discussion by multiple mothers.

*“And definitely the weight and scale nursing was so vital for my son. And the virtual visit, there's no weight for the baby”* Mother in BabyCare program.

*“Like she said the virtual visits were okay, but you weren't able to get the weights like the weight of the baby is what we usually do in the in-person visits, which if you know your baby's not gaining as much, it would've been harder to find out unless you actually go to the doctor and. By then. It's not as often that you’re going to the doctors. You're going to see or hear a nurse in the middle of going to your doctor. So that was really helpful for me. So I would also prefer the in-person visits“* Mother in BabyCare program.

iii) IPHV is the only way to have a comprehensive assessment of the family and environment around both the baby and the mother. Some nurses attributed this to the way in which they received their training in nursing, which emphasized using all senses while walking in the room. The nurses described with examples how IPHV is essential to provide a whole assessment of the child, family, and home environment. As an example, in her experience one nurse spotted a leaky ceiling that was about to fall, and another spotted a fire hazard. As one of the nurses put it “There's one thing too, is that you can't smell gas if you're on the phone.”. Another nurse described the importance of gathering information from the area outside the house during IPHV. Another example of how IPHV may bring unexpected benefits to mothers is that the nurse noticed during home visit that the mother was throwing away ripe bananas that she obtained through the food bank. She detailed how she taught the mother how to make several recipes from a ripe banana.

*“So, like I said, I did home visiting in the past and I've learned the value of being able to go into a home, and see for yourself, a whole assessment of not just the baby and the family, but the home environment itself. And, things were kind of dangerous were presented where I have seen one with like a leaky ceiling that was actually with a piece of Plexiglas over the bathtub where mom would bathe the kids. And it actually looks like it was about to fall and crash. So, there was that, I never would've seen through Zoom itself.”* Nurse in BabyCare.

*“I know everybody's hit on why it's so important that we go into the home and how much we learn. But I will say I also learned a lot as a home visitor by my drive, into the area in which they live, you start your assessment there and you really look at, are there doctor's offices within walking distance? Is there a grocery store within walking distance? What does the housing area look like that they live in? Does it look safe from a physical standpoint?”* Nurse in BabyCare*.*

Mothers also saw the potential benefits of IPHV in detecting abnormal health conditions in their children. Several mothers gave specific examples of how the interaction during IPHV resulted in a benefit including, for example a referral of children to physical therapy early for developmental issues and solving several issues related to breastfeeding. Some African American mothers described how the interaction with the nurses during IPHV helped them to continue exclusive breastfeeding for their children despite the pressure from their mothers-in-law to feed the child formula milk.

*“I mean definitely, it's really hard because my kids are both developmentally delayed, just, I guess, bad genetics. And a lot of things I worry about, I don't feel like she could see with her phone because seeing a picture of something and actually seeing something in-person is like two totally different things.”* Mother in BabyCare program.

*“Um, she gets to see my baby. I mean, see things that I don't see. You know, she knows what she'... You know, as a nurse, she knows what to look for, in certain cries or in certain movements, you know certain things that she should be doing, even not be doing.”* Mother in BabyCare program.

*“Also, when I did that in person, she was able to say, you know, I can hear your sons nursing is a lot louder so let's go ahead and get consulted, those things. You know, if you don't know any better and there's nobody there and you're just like well, my kid just can't nurse. You wouldn’t know, but the nurse knows what to look for and to listen for, to really put in place things that can help”* Mother in BabyCare program.

*“My mother-in-law is like, "Oh, the baby is hungry. The baby is not feeding well now." Oh, my gosh. The baby is feeding well! She took Similac and was gonna feed my baby. I'm like, "No, I wanna do six months exclusive. So, when the nurse came, the nurse started demonstrating the size of the baby for her.”* Mother in BabyCare program.

iv) IPHV allows knowing people better and that they may open up and tell or share with the nurse clues or information. One of the nurses stated *“What I like about in-home visiting is you get to know your people so much better. They will open up to you and tell you things they might wouldn't tell you if you don't have that in person connection.”*. Nurses suggested that IPHV is essential to collect clues and establish a connection to get valid information on sensitive issues like domestic violence. As one of the nurses said, *“.. history of domestic violence, who is going to admit that the first time you see someone? but if you're in the home, sometimes you can get a feeling if there is or not”*.

The nurse shared her experience with a mother who asked whether her husband had to be available during IPHV. A few minutes after the nurse arrival at home, the husband, who was supposed to be at work, arrived and took the baby and answered any question the nurse asked. Later, it was found that the husband was extremely abusive, and the mother had to be protected from domestic violence with the help of the nurse. Mothers also reported that IPHV helps them to open up more and express themselves. As Spanish speaking mother said, *“Well, as the lady said one opens more and yes, one expresses themselves more.”.*

e) IPHV is essential for social and emotional support as well as for developing a better connection.

Nurses thought that IPHV is essential for mothers who do not have family members as the nurse can be the only person who visits them. As explained by a nurse, *“one of the reasons is because sometimes our clients don't have family. So, we can be the person that they have, that they can feel like they have someone, that's gonna come, visit with them, talk with them. Sometimes you go and do your visit and you get everything that has to be done for BabyCare out the way. And then they just want to talk to someone and have someone there with them because you may be the only person that they're gonna have in their home for weeks, months. So, I do prefer in home.”.* This seems to be a major issue from the mothers’ perspective, who described their nurse as a family member several times in different group discussions highlighting that IPHV creates a better connection not only between them and the nurse but also with their children. Some mothers kept the connection with their nurses even after their children are grown and no longer in the program.

*“Um, I like the connection, the bond yeah…. more person, more personal, you know, get to, interact with each other way more. You know, my kids created obviously a bond. I have a bond with her. It's more bonding. I feel like in-person, you know, virtually, yes, it's okay, but I'd prefer in-person all day, every day due to the fact it is like you, that's how I could have relationship, a relationship with her. I have relationship with her.”* Mother in BabyCare program.

*“But yes, did we missed her coming around because I was so used to her coming around? My kids are so used to her coming around and being around her. Yes, I feel like not our relationship had like distance, but it just wasn't like how it used to be, for being so long. You understand what I'm saying?”* Mother in BabyCare program.

*“Now, I would prefer that in-person as well. So, I've had so my son was very attached to her and he would run up and hug her, get all excited when I told him she's coming and visit us where it's my daughter its virtual so she's like hey how you doing and then just run off.”* Mother in BabyCare program.

*“I'd like to add that I hope that they keep it in person and everything like that because like. We built bonds with the nurses as well. My first nurse that I have, my five-year-old. I ended up talking to her a year after my daughter aged out. Just regularly checked up with her. And she was genuinely like interested in how my daughter was doing everything then of course she retired.”* Mother in BabyCare program.

Mothers during groups discussion showed their excitement that IPHV would be resumed and that they missed a lot through VHV. Of note, none of the Spanish speaking mothers described nurses as a family member but highlighted that they gained confidence in them and considered them as friends. While Spanish speaking mothers did not have a strong preference for IPHV, some mothers preferred IPHV for better understanding of English when talking to the nurse in-person.

*“It seems to me, for me it is fine in person and on the phone. No problems because they treat me the same way. How do I tell you, that they do provide such good services, but when they look for a translator, you can't say blah blah blah, just one word, that is, no, you can't talk much because you're just like you are like this here and so it happens yes or no? And a small phrase, of course, is not the same as in person. If we could speak about 60% or 70% of Spanish, would we be able to communicate, even by phone or wherever. Yes, of course, although they do look for a way to treat us well or find a way to communicate, but I feel that it is not the same. I want in-person.”* Spanish speaking Mother in BabyCare program.

*“She treats us all well, everything well. But I would like, for example, if it continued like this with the virtual visits. I would like someone who speaks Spanish so that we can understand each other much better.”* Spanish speaking Mother in BabyCare program.

### Positive and negative aspects of virtual home visits

One obvious advantage of VHV that nurses have cited repeatedly is the flexibility it offers for busy mothers, despite the difficulty to establish a relationship. Although not in favor of VHV, Spanish speaking mothers highlighted the advantage of VHV as it is possible to receive VHV while visiting friends or wherever they were. Some mothers accepted VHV because it was safe despite that it was not their choice. A few mothers also thought that VHV is easier as they do not have to make their living room clean before the nurse visit.

The nurses also highlighted the difficulty of VHV when the mother or the caregiver has intellectual disabilities. As one of the nurses expressed her concern, *“The only concern I have with virtual visit is regarding when we have CPS referrals or if we have moms with intellectual or caregivers with intellectual needs. I think it's more challenging on a virtual visit. I think home visiting definitely is we should be doing that with our CPS cases as well.”*. This particular point was raised by a mother who has child with special needs and reported that VHV is hard for her and her child. A mother of child with special needs said, *“I do have to say it's not with BabyCare specifically, but the virtual visits were really hard. So, my son has special needs, and he has to see faces like right there. And even with school, virtual was just hard overall. I mean he has to see the face. He has to touch people. He has to kind of get used to people, and it feels like talking to a stranger, right? Talking on the phone”*

One of the nurses stated that one of the barriers to VHV in this vulnerable group is the inability to pay their phone bills while another nurse found that VHV is not the best way for mothers to see her enthusiasm and passion. Some mothers described how difficult to show the child through a video camera while it would be much easier if they had IPHV. Finally, among Spanish speaking mothers, there was also a preference for IPHV but more acceptance for VHV. As Spanish speaking mother summarized, *“Ah, well I think it's better in-person. But this way is fine too”.*

### Video or audio home visiting preference

When it comes to VHV, nurses preferred video conference instead of telephone calls/audio only. Nurses asserted that video conference allow for some assessment beyond what could be done in an audio only visit. As one of the home visitors explained, *“I feel like, body language contributes to a lot. And I know like when I first got hired, we talked about like, how can we assess the environment. It still puts eyes in the home regardless. So, I still feel like I could gain knowledge, that I normally wouldn't just get from a phone call with a family. I mean, again, it still presents challenges and a lot of limitations, but in comparison to a phone call that we may have been restricted to, if we didn't have the technology available to us, I feel like it was so important in this aspect. And at least to gain some kind of home visiting experience”*. As mentioned above, the supervisor nurse cited examples of VHV in which assessment of developmental milestones was conducted through video conferencing. Mothers also felt that some assessment of developmental milestone is possible with video VHV but not audio VHV clearly showing their preferences for video VHV. As one mother explained *“But with the video, she actually seeing the baby. She's able to not just talk to me, but she's interacting also with him. So, I think I like the video versus the audio”* while another mother stated, *“Because they can see the baby more. You know it's all about the baby. Not, not about me. It's about the baby. And that's what I'm concerned more with”*. Only one mother preferred audio VHV because she found it difficult to make her children sit in front of the camera even for a few minutes, therefore, she preferred to describe to the nurse what her children can or cannot do. Nurses asserted that mothers and children are usually existed to see someone through the video compared to audio visits. One of the nurses said, *“I can see the difference when I home visit my clients now from a distance, you know, they just so excited to see someone, you know, instead of over the phone”*. This was supported by mothers who reported that their children like to see the nurse through the video. One mother said, *“And my son always liked waving at the camera. So, the audio call can’t see the kid”*

### Prospective role of Virtual Home Visiting (VHV)

Despite the strong preference for IPHV, nurses see a great value in keeping the option of VHV based on circumstances. As mentioned earlier, nurses felt that busy mothers are comfortable with VHV. The nurses also suggested that assessment of developmental milestones virtually could be improved if they receive training on conducting the assessments virtually.

*“I really do hope that they will choose to let us use our discretion, the nurses discretion as to what is gonna be better. Because I think of the times that I was a case manager and home visitor, and we would have to cancel because mom or baby were sick and you could have the potential option to, well, can I, can we just go ahead and switch to virtual and still accomplish what we needed to accomplish for the most part. So, I would like the flexibility to do both.”* A supervisor nurse in BabyCare program*.*

*“I think it would be very nice if the department of health provided training for this type of virtual visiting. Because as it was said here, there are a lot of things cannot be done, but there is still a lot that can be done virtually that maybe we're missing because we don't, maybe we're just missing because we don't know that can be done virtually. So, it would be nice to have not only the technological training, but also as outreach workers or nurses, what can be done virtually or what can be assessed or what can be taught.”* A nurse in BabyCare program.

Mothers were excited when they heard from their home visitors that IPHV will be resumed shortly. One mother said, *“But now they are starting to do home visits again the month of April, so I can't wait to see her”.* When the facilitator asked what if it is postponed many mothers were disappointed arguing that IPHV should be resumed because home visitors are fully vaccinated, always check their temperature, and wear masks. As one of the mothers said, *“They go through rigorous, you know, probably COVID vaccines and COVID testing. And I'm sure their bosses and their bosses' bosses are swabbing and temperature checking every day. So, I feel like they have enough precautions in place that we can safely start doing it.”.* In one group discussion, some mothers indicated that families would not be interested to be part of the program if IPHV is totally abandoned. One mother said, *“So, I feel like taking that piece away may make people not want to be a part of the program”.* While in other group discussion, mothers thought that having only VHV would defeat the purpose of the program. As one mother explained, *“I think it defeats the purpose of the BabyCare Program. Yeah, that's my feeling. Because I can remember when she comes, she takes measurements of the baby. She takes the weight. How you gonna do that visually?”.*

## Discussion

Our finding showed that both home visitors and mothers envisage IPHV as a superior to VHV for multiple reasons. Home visitors believed that IPHV is the way to have a comprehensive assessment for the child, family, and the environment inside and outside the house presenting several real-life examples based on their work experience. Both home visitors and mothers considered IPHV critical for proper assessment of child developmental milestones as well as physical growth, despite that both mothers and home visitors acknowledged that some assessment is possible through video VHV. Home visitors suggested that more training on the assessment of developmental milestones virtually may help resolve some issues related to the assessment using VHV. The Rapid Response to VHV collaborative provided technical assistance and uidance related to VHV service delivery, which meant to meet the needs of all home visiting staff delivering all MIECHV models [27]. In fact, VHV workforce training protocols were developed even before the onset of COVID-19 [5]. Also, several organizations have developed training protocols for different professionals on remote assessment of the child development outside the context of MIECHV [28, 29]. Many of these organizations still encourage the live assessment and some found that training is necessary not only for home visiting staff but also for the parents to help with the assessment. Even if a reliable virtual assessment method is developed and the training for home visiting staff is conducted, parents’ perceptions related to the effectiveness of virtual assessment needs to be improved. Our findings also showed that home visiting staff consider live assessment of milestones as an enjoyable part of their job and as an opportunity to build the relationships with the families.

Home-visitors reported several instances in which IPHV produced several benefits resulting from the interaction between the family and the home-visitor. As an example, IPHV can help detecting clues of domestic violence, which are likely to be overlooked in VHV. A recent systematic review and meta-analysis found that reports of suspected domestic violence increased during the pandemic due to stay-at-home and lockdown orders [30]. Although this is supposed to lead to a higher number of cases of depression, a recent report showed that referral from MIECHV programs to mental health has declined during COVID-19 [5], which could be due the failure to detect cases of depression and clues of domestic violence through VHV.

One of the major findings in our study is that despite mothers appreciate that the program covered their material needs, most mothers focused on their personal relationship with the nurse, with mothers describing their nurse as part of their family. Mothers viewed IPHV as an essential way to optimally engage with home visitors hence build the relationship between them and their home visitor. It is hypothesized that the evidence-based models of MIECHV through their consistency, structure, and focus on achieving the program outcomes, divert the attention from (or sometime conflict with) building home visitor–parent relationships [31–33]. In a study in Illinois in 2014, home visitors felt that their clients were being treated as “numbers” and “result” rather than seen as human beings facing and overcoming everyday obstacles and reported their concerns that paperwork during each home visit interrupted the natural course of relationship building with their clients, diminishing the client-centered nature of the program [33]. Even before the pandemic, home visitors in MIECHV in Florida felt that their personal connection with the families was not given the same level of importance as the outcome data that required documentation [34]. A recent literature review of parents’ satisfaction with sustained home visiting care for mothers and children at global level, showed that nurse-client relationship is a critical factor for parents’ satisfaction [22]. It has been also demonstrated that the success of home visiting interventions to great extent depends on home visitor’s ability to develop therapeutic relationship with the client [35]. Our findings suggest that from both home visitors and parents’ perspectives, VHV may further weaken building home visitor–parent relationships in MIECHV programs.

Several mothers felt that relying heavily or exclusively on VHV may defeat the purpose of the home visitation and that some families even may refuse to be part of the program. This supports previous assertion that home visiting programs may not be able to effectively engage families through VHV [5]. Both home-visitors and mothers see that VHV has a role to play if mothers are busy. Previously, in the MinuteClinic telehealth pilot program, mothers who were often busy with work, childcare, and other responsibilities preferred telehealth rather than access to traditional medical care [36].

Our findings suggest that using VHV with parents, who have intellectual disabilities is not recommended as both home visitors and mothers reported that VHV is challenging in this case. Overall, there is a great need to understand and strengthen MIECHV services for parents with intellectual disability [37]. Finally, the fact that none of the mothers during group discussions raised the issue of difficulty in using technology as a major issue in VHV is worthy of note. Several explanations can be proposed including that the participants may have gained the experience and no longer have difficulty with dealing with the technology. Similarly, none of the mothers stated their inability to pay the internet bill although home-visitors raised this issue.

This study has several strengths including exploring both the mothers’ and home visitors’ perspectives. One of the limitations of this study is conducting this study only in one of the MIECHV and that findings of this study may not be extrapolated to other programs. However, this work will pave the way to study mothers’ and home visitors’ perspective on VHV in other MIECHV programs in Virginia.

## Conclusion

Families enrolled in MIECHV program had unique needs at the onset of pandemic attributed to job loss and children are out of school and childcare [38], which may have changed now. Also, both mothers and home visitors have gained experience with VHV, and it is possible that their experience and perceptions are now different from their experience at the beginning of the pandemic. We explored the current experience and perceptions of both home visitors and mothers on VHV in one of the MIECHV programs in Virginia. We found that mothers and nurses see IPHV to be critical for proper and comprehensive assessment of the child and the family and also essential to build the nurse-client relationship. Home visitors believe that IPHV can help detecting clues of domestic violence, which are likely to be overlooked in VHV. Both mothers and home visitors see VHV as a supplementary to IPHV that can be used from time to time particularly with busy mothers. Our findings also showed that VHV may have little room with parents with intellectual disabilities. Finally, if VHV is to be used, both mothers and nurses prefer video rather than audio only, and difficulty in dealing with technology seems to be no longer a major issue.

### Supplementary Information

**Additional file 1.**

## Data Availability

The transcripts of the focus groups discussion analyzed in this study are available from the corresponding author on reasonable request.

## Abbreviations

MIECHV: Maternal, Infant and Early Childhood Home Visiting
VHV: Virtual Home Visiting
IPHV: In-person Home Visiting

## References

[1] HRSA (2021). The Maternal, Infant, and Early Childhood Home Visiting Program. Health Resources and Services Administration.

[2] Michalopoulos C, Faucetta K, Warren A, Mitchell R (2017). Evidence on the long-term effects of home visiting programs: Laying the groundwork for long term follow-up in the Mother and Infant Home Visiting Program Evaluation (MIHOPE).

[3] Models eligible for Maternal, Infant, and Early Childhood Home Visiting (MIECHV) funding [https://homvee.acf.hhs.gov/HRSA-Models-Eligible-MIECHV-Grantees].

[4] Important Home Visiting Information During COVID-19 [https://mchb.hrsa.gov/Home-Visiting-Information-During-COVID-19].

[5] Traube DE, Molina AP, YingWangKay S, Kemner A (2022). Perinatal mental health support and early childhood home visitation during COVID-19. Prev Sci.

[6] Duryea EL, Adhikari EH, Ambia A, Spong C, McIntire D, Nelson DB (2021). Comparison between in-person and audio-only virtual prenatal visits and perinatal outcomes. JAMA Netw Open.

[7] Holcomb D, Faucher MA, Bouzid J, Quint-Bouzid M, Nelson DB, Duryea E (2020). Patient perspectives on audio-only virtual prenatal visits amidst the severe acute respiratory syndrome coronavirus 2 (SARS-CoV-2) pandemic. Obstet Gynecol.

[8] Liu CH, Goyal D, Mittal L, Erdei C (2021). Patient Satisfaction with Virtual-Based Prenatal Care: Implications after the COVID-19 Pandemic. Matern Child Health J.

[9] Marshall J, Kihlström L, Buro A, Chandran V, Prieto C, Stein-Elger R, Koeut-Futch K, Parish A, Hood K (2020). Statewide Implementation of Virtual Perinatal Home Visiting During COVID-19. Matern Child Health J.

[10] The Crucial Role of Home Visiting During COVID-19: Supporting young children and families [https://www.chcs.org/the-crucial-role-of-home-visiting-during-covid-19-supporting-young-children-and-families/].

[11] Hall JA, Roter DL, Katz NR (1988). Meta-analysis of correlates of provider behavior in medical encounters. Med Care.

[12] Shirley ED, Sanders JO (2013). Patient satisfaction: Implications and predictors of success. J Bone Joint Surg Am.

[13] Kim SS, Kaplowitz S, Johnston MV (2004). The effects of physician empathy on patient satisfaction and compliance. Eval Health Prof.

[14] Chou CY, Brauer DJ (2005). Temperament and satisfaction with health status among persons with rheumatoid arthritis. Clin Nurse Spec.

[15] Grosset KA, Grosset DG (2005). Patient-perceived involvement and satisfaction in Parkinson's disease: effect on therapy decisions and quality of life. Mov Disord.

[16] Ware JE, D.avies-Avery A, Stewart AL (1978). The measurement and meaning of patient satisfaction. Health Med Care Serv Rev.

[17] Pascoe GC (1983). Patient satisfaction in primary health care: a literature review and analysis. Eval Program Plann.

[18] Ware JE, Davies AR (1983). Behavioral consequences of consumer dissatisfaction with medical care. Eval Program Plann.

[19] Ho PS, Stegall MB, Wan TT (1994). Modeling two dimensions of patient satisfaction: a panel study. Health Serv Manage Res.

[20] Fitzpatrick R (1991). Surveys of patients satisfaction: I-Important general considerations. BMJ.

[21] Larsen DE, Rootman I (1976). Physician role performance and patient satisfaction. Soc Sci Med.

[22] Kanda K, Blythe S, Grace R, Kemp L (2022). Parent satisfaction with sustained home visiting care for mothers and children: an integrative review. BMC Health Serv Res.

[23] Donabedian A (2005). Evaluating the quality of medical care. 1966. Milbank Q.

[24] Mahon PY (1996). An analysis of the concept 'patient satisfaction' as it relates to contemporary nursing care. J Adv Nurs.

[25] BabyCare Fact-Sheet [https://www.vdh.virginia.gov/central-virginia/babycare/].

[26] Patton MQ (2002). Qualitative Research and Evaluation Methods.

[27] Rapid Response Virtual Home Visiting [https://rrvhv.earlyimpactva.org/].

[28] Can you conduct a reliable child assessment, remotely? [https://www.gesell-yale.org/blogs/sparking-wonder/can-you-conduct-a-reliable-child-assessment-remotely].

[29] Meaningful Assessment [https://www.virtuallabschool.org/infant-toddler/program-management/lesson-3/act/14876].

[30] Alex RP, Wesley GJ, Erin J, Catherine K, Felicia Marie K (2021). Domestic violence during the COVID-19 pandemic - evidence from a systematic review and meta-analysis. J Crim Just.

[31] Sheila JB, Jean Ann S, Kathy RT, Jean MI, Valeri JL (2006). Building successful home visitor–mother relationships and reaching program goals in two early head start programs: a qualitative look at contributing factors. Early Child Res Q.

[32] Dunst CJ, Boyd K, Trivette CM, Hamby DW (2002). Family-oriented program models and professional helpgiving practices. Fam Relat.

[33] Barak A, Spielberger J, Gitlow E (2014). The challenge of relationships and fidelity: home visitors' perspectives. Child Youth Serv Rev.

[34] Alitz PJ, Geary S, Birriel PC, Sayi T, Ramakrishnan R, Balogun O, Salloum A, Marshall JT (2018). Work-Related Stressors Among Maternal, Infant, and Early Childhood Home Visiting (MIECHV) home visitors: a qualitative study. Matern Child Health J.

[35] Kitzman H, Olds DL, Henderson CR, Hanks C, Cole R, Tatelbaum R, McConnochie KM, Sidora K, Luckey DW, Shaver D (1997). Effect of prenatal and infancy home visitation by nurses on pregnancy outcomes, childhood injuries, and repeated childbearing. A randomized controlled trial. Jama.

[36] Polinski JM, Barker T, Gagliano N, Sussman A, Brennan TA, Shrank WH (2016). Patients' satisfaction with and preference for telehealth visits. J Gen Intern Med.

[37] West AL, Dibble KE (2022). Evidence-based early home visiting for mothers and parents with intellectual disability: home visitor perceptions and practices. Intellect Dev Disabil.

[38] Cluver L, Lachman JM, Sherr L, Wessels I, Krug E, Rakotomalala S, Blight S, Hillis S, Bachman G, Green O (2020). Parenting in a time of COVID-19. Lancet.

