# Supplementary material for: Virtual home visits during COVID-19 pandemic: mothers’ and home visitors’ perspectives
Source: BMC Pregnancy Childbirth. 2023 Aug 11;23:577. doi: 10.1186/s12884-023-05896-9 (PMC10422766; doi:10.1186/s12884-023-05896-9)
Supplement: Supplementary file 1 — Additional file 1. [file 12884_2023_5896_MOESM1_ESM.docx]

**List (1a): of codes from group discussion with staff at BabyCare program.**

| **1. Motivation to work in the program:** | |
| --- | --- |
| 1.a | The program involves going to the community and meet, connect, and engage/interact with mothers and families. |
| 1.b | Previous experience that inspired nurse to work in the program including working in schools, nursey, and hospitals |
| 1.c | Playing with babies and assessing their milestones and talking to the parents |
| 1.d | What the program provides to children and mother as a solid help. |
| 1.e | Working with families as a whole including extended families |
| 1.f | Feeling that people trust them and welcome them in their house. |
| 1.g | See what children and families have accomplished with the program |
| 1.h | The nature of the job requires switching gears from home visiting to working in the office to coordinating with other agencies. |
| 1.i | Work in the program is challenging |
| 1.j | Workplace support from colleagues and having great support staff. |
|  |  |
| **2. Importance of in-person home visit for the mission of the programs:** | |
| 2.a | Necessary for proper assessment of the developmental milestones for children. |
| 2.b | Necessary for the assessment of the growth of children (measuring the weight and sometimes height) |
| 2.c | Essential to have a comprehensive assessment of the child, family and environment around the children/mothers inside and outside the house. |
| 2.d | Allow knowing people better so that they may open up and tell or share with the staff essential clues or information on sensitive issues like domestic violence. |
| 2.e | Essential for mothers who do not have other family members |
|  |  |
| **3. Positive & negative aspects of virtual home visits:** | |
| 3.a | Offers flexibility for busy mothers |
| 3.b | Need attention to the wording and communication particularly during assessment |
| 3.c | Difficult when dealing with mothers or caregivers with intellectual disability. |
| 3.d | Requires abilities to pay the phone/internet bills. |
|  |  |
| **4. Video or Audio home visiting preference:** | |
| 4.a | Video virtual home visiting allows for more assessment beyond what can be done in an audio only home visiting. |
| 4.b | Some mothers are existed to see their nurses through the screen. |
|  |  |
| **5. Future of VHV staff and mother prospective:** | |
| 5.a | Have VHV as an option to be conducted based on circumstances and discretion of the nurse |
| 5.b | Conduct training to improve the skills of child assessment virtually. |
|  |  |

**List (1b): codes from groups discussion with mothers enrolled in BabyCare program.**

| **1.Experience with program in general:** | |
| --- | --- |
| 1.a | Describing the program in positive terms: really helpful, awesome, meet our needs, etc. |
| 1.b | Describing staff working with the program in positive terms: very sweet and very caring, nonjudgmental, advocate for me, awesome, goes above and beyond. |
| 1.c | Describing staff working with the program as a family member. |
| **2. Services provided by the program and highly appreciated by mothers:** | |
| 2.a | Providing material needs such as car seat, clothes, shoes, food, formula (during formula shortage), books, connecting mothers to sources of help, baby monitors, set up playpen, breast pumps, etc. |
| 2.b | Providing emotional and social support to mothers and children. |
| 2.c | Providing a second opinion in medical conditions or guidance to proper medical care. |
| 2.d | Assessment of developmental milestones for children |
| 2.e | Assessing and monitoring of the growth of children |
| 2.f | Helping with job search for mothers |
| 2.g | Help with breastfeeding and other feeding issues |
| **3. Importance of in-person home visiting:** | |
| 3.a | Essential for assessing developmental milestones for children |
| 3.b | Essential for the assessment of the growth of children (measuring the weight) |
| 3.c | Important to create better relationship and bonding between mothers and staff as well as between staff and children. |
| 3.d | Several benefits from the nurse assessing the child (or mother) during the interaction Example 1 to Example 6. |
| 3.e | Results in better communication to solve linguistic barrier (Spanish speaking mothers) |
| 3.f | Help mothers to open up and express themselves more |
| **4. Positive and negative aspects of virtual home visiting** | |
| 4.a | Description of virtual home visiting in general as hard |
| 4.b | Difficult when dealing with mothers or caregivers with intellectual disability. |
| 4.c | Difficult to show the child through the camera particularly very young children |
| 4.d | Mothers’ perception about the risk during pandemic increased their acceptance for VHV |
| 4.c | VHV saves gas |
| 4.e | VHV is safe |
| 4.f | VHV provides flexibility for mothers who are on the go or visiting friends |
| 4.g | VHV is less burdensome as it does not require preparation of living room to look clean as with what mother do before IPHV |
|  | |
| **5. Future of home visiting** | |
| 5.a | Mothers waiting and expecting IPHV to be resumed. |
| 5.b | Some mothers can see role for VHV |
| 5.c | Some mothers may refuse to be part of home visiting program if IPHV is abandoned. |
| 5.d | Some mothers see using VHV only defeat the purpose of the home visiting programs. |
|  |  |
| **6. Video or Audio preference:** | |
| 6.a | Video VHV allows for some assessment beyond what can be done in an audio only visit. |
| 6.b | Some kids are existed to see someone through the screen. |
